# Artistic-Value Descriptions Are Associated with Higher Aesthetic Ratings than Social-Value Descriptions for AI-Generated Variants of Classical Paintings

**DOI:** 10.3390/bs16081437

**Published:** 2026-08-20

**Authors:** Qi Li, Qi Li, Chaoyuan Zhang, Ziyu Feng

**Affiliations:** School of Fine Arts, Shanxi University, Taiyuan 030006, China; liqi27@sxu.edu.cn (Q.L.); zhangchaoyuan@sxu.edu.cn (C.Z.); fengziyu@sxu.edu.cn (Z.F.)

**Keywords:** aesthetic cognition, empirical aesthetics, art perception, contextual information, AI-generated images, value cues, mixed-effects model

## Abstract

Contextual meaning can influence art evaluation even when viewers encounter images within a structured visual stimulus family. This exploratory questionnaire-version study examined whether value-oriented textual descriptions were associated with aesthetic ratings of AI-generated variants of classical paintings in three Chinese-language questionnaire versions emphasising cultural value, artistic value, or social value. The available dataset contained 61 participants, each rating 40 images on a 1–10 aesthetic scale, yielding 2440 complete observations. A post hoc three-rater manipulation check of 30 image–description pairs found that all evaluated pairs satisfied the applied validation criterion; inter-rater reliability across item–dimension judgments was high at ICC(2,k) = 0.936. The primary analysis used a linear mixed-effects model with crossed random intercepts for participant and stimulus identity, and a fuller by-stimulus random-slope model was estimated as a diagnostic response to review. Relative to social-value descriptions, artistic-value descriptions were associated with higher ratings in the random-intercept model, where b = 1.74, SE = 0.57, z = 3.03, *p* = 0.002, 95% CI [0.62, 2.87], and this contrast also persisted in the diagnostic by-stimulus random-slope model, although that model converged with a non-positive-definite Hessian. Cultural-value descriptions were intermediate and should not be interpreted as showing a stable general advantage over social-value descriptions. The results therefore provide qualified evidence for an artistic-versus-social framing association, not a confirmed causal effect of all value-cue conditions. Interpretation remains bounded by the modest sample, undocumented allocation procedure, rapid-completion records, incomplete prompt metadata, and post hoc validation of a representative rather than exhaustive stimulus set.

## 1. Introduction

Aesthetic appreciation is not adequately described as a direct readout of visual properties. Information-processing accounts describe an aesthetic episode in which perceptual analysis interacts with memory, classification, understanding, emotion, and evaluation (Leder et al., 2004; Leder & Nadal, 2014). The Vienna integrated model further situates appraisal within reciprocal bottom-up and top-down processing, allowing contextual knowledge to alter the trajectory from perception to judgement (Pelowski et al., 2017). Neuroaesthetic reviews likewise indicate that aesthetic valuation involves distributed perceptual, affective, and reward-related systems rather than a single response to image form (Chatterjee & Vartanian, 2014; Skov, 2019). Consequently, an image may be evaluated differently when it is encountered as evidence of technique, as a cultural symbol, or as a representation of social meaning.

A related explanation concerns processing fluency: an interpretation that makes salient features easier to organise or understand may contribute to positive affect and liking (Forster et al., 2013; Reber et al., 2004). Fluency alone is not a complete account of art reception, because historically and personally situated meanings also matter. The psycho-historical framework proposed by Bullot and Reber emphasises that appropriate appreciation can require knowledge of an artwork’s production, functions, and historical relations (Bullot & Reber, 2013). Together, these approaches predict that a textual description can affect judgement through more than one route: it may draw attention to perceptually available form, activate cultural knowledge, or articulate social–historical significance.

Evidence that verbal and environmental context changes art reception is now substantial. Meaningful titles can increase understanding and enjoyment of ambiguous artworks (Millis, 2001). Descriptive or biographical information changes appreciation of established paintings (Swami, 2013), while semantic context modulates aesthetic value and corresponding neural responses (Kirk et al., 2009). More recently, contextual information has been shown to affect aesthetic engagement across visual artworks (Darda & Chatterjee, 2023). The broader encounter context also matters: museum presentation and viewing setting can alter viewing time, valuation, and memory relative to laboratory-like exposure (Brieber et al., 2014, 2015). These findings make contextual framing an experimentally tractable component of aesthetic cognition, but they do not establish that every type of context has the same influence.

The use of AI-assisted imagery creates a timely setting to test semantic context. Research on computer-generated art predates recent text-to-image systems and has shown that viewers can respond aesthetically to algorithmic images (Chamberlain et al., 2018). At the same time, philosophical and curatorial analyses have raised questions about agency, intention, and creativity in computer-assisted production (Hertzmann, 2018; Mazzone & Elgammal, 2019). Empirical work after the emergence of generative systems has focused mainly on authorship information: human-created labels may raise preferences or judgements of creativity relative to AI-created labels (Bellaiche et al., 2023; Demmer et al., 2023; Horton et al., 2023); implicit responses may also reveal attitudes toward AI art (Zhou & Kawabata, 2023), and self-relevance remains predictive of appeal for real and synthetic artworks (Vessel et al., 2023). Individual experience and attitudes further help explain negative bias toward AI-generated art (Grassini & Koivisto, 2024). Recent studies published after the present data were collected continued to find that AI-versus-human labels can influence aesthetic judgement, including in lay-viewer and Chinese-viewer samples (Lin et al., 2026; Wang et al., 2026). Generative AI therefore matters both as a stimulus-production technology and as a possible source of framing effects (Epstein et al., 2023).

The present study addresses a narrower and distinct question. It does not compare images labelled as made by humans or by AI. Instead, it examines whether semantic value cues attached to AI-generated visual variants shift aesthetic ratings. Three dimensions were operationalized in the questionnaire. Cultural-value cues emphasised tradition, symbolic meaning, and cultural identity; artistic-value cues emphasised technique, composition, colour, and execution, and social-value cues emphasised social background, public relevance, class, or everyday life. These dimensions represent framing conditions, not complete definitions of the intrinsic value of an artwork.

The selection of cultural-, artistic-, and social-value cues was grounded in three overlapping but conceptually distinguishable routes through which context can enter aesthetic judgement. Cultural-value cues refer to symbolic tradition, shared memory, and historically transmitted meaning, aligning with psycho-historical accounts of art appreciation in which knowledge of origin, function, and cultural relations can shape evaluation. Artistic-value cues refer to formal execution and perceptually available craft, such as composition, colour, light, and detail, aligning with models of perceptual organisation and processing fluency. Social-value cues refer to public relevance, social background, class, role relations, or everyday-life significance, aligning with contextual accounts in which an artwork is evaluated as a socially situated object. These categories were therefore treated as framing dimensions rather than as mutually exclusive ontological properties of the artworks.

The study contributes in three qualified ways. First, it extends research on contextual information from titles and authorship labels to descriptions that foreground different value dimensions. Second, it uses repeated stimulus families derived from five canonical paintings, enabling cue-condition differences to be examined while acknowledging recurrent stimulus identity. Third, in response to editorial concerns about manipulation validity, it reports a post hoc three-rater manipulation check for representative image–description pairs and explicitly limits interpretation to the documentary record available for the present revision.

The following exploratory directional expectations were evaluated in the available questionnaire data: E1, aesthetic ratings differ across description-cue versions; E2, cultural-value descriptions receive higher ratings than social-value descriptions; and E3, artistic-value descriptions receive higher ratings than social-value descriptions. Because the supplied records do not document preregistration or a prospectively time-stamped analysis plan, these are reported as exploratory expectations rather than preregistered confirmatory hypotheses.

## 2. Materials and Methods

### 2.1. Design and Participants

The study used three between-participant questionnaire versions differentiated by description-cue condition: cultural value, artistic value, and social value. The evidence available for the present revision consisted of three SPSS (IBM SPSS Statistics 27.0) files exported from the Wenjuanxing questionnaire platform. Condition membership was determined by the version-specific export file. After merging the exports, the analytic dataset contained 61 completed participant records: 18 in the artistic-value condition, 21 in the social-value condition, and 22 in the cultural-value condition. Each record contained ratings for 40 image–description items, yielding 2440 non-missing aesthetic-rating observations. Because the exported files do not document allocation mechanism or recruitment flow, the design is treated in this revision as an exploratory questionnaire-version comparison with limited causal status rather than as a fully documented randomised experiment.

The outcome variable was the rating of image aesthetics on a 1–10 scale, where higher values indicated greater perceived beauty or aesthetic appeal. The supplied exports establish the number of completed records and item scores. The available records do not allow independent verification of recruitment channel, eligibility criteria, participant art expertise, whether each person could access more than one questionnaire version, or whether item presentation order was randomised. These missing procedural details are treated as limits on internal validity, causal interpretation, and reproducibility.

### 2.2. Stimuli and Value-Cue Manipulation

**Figure 1:**
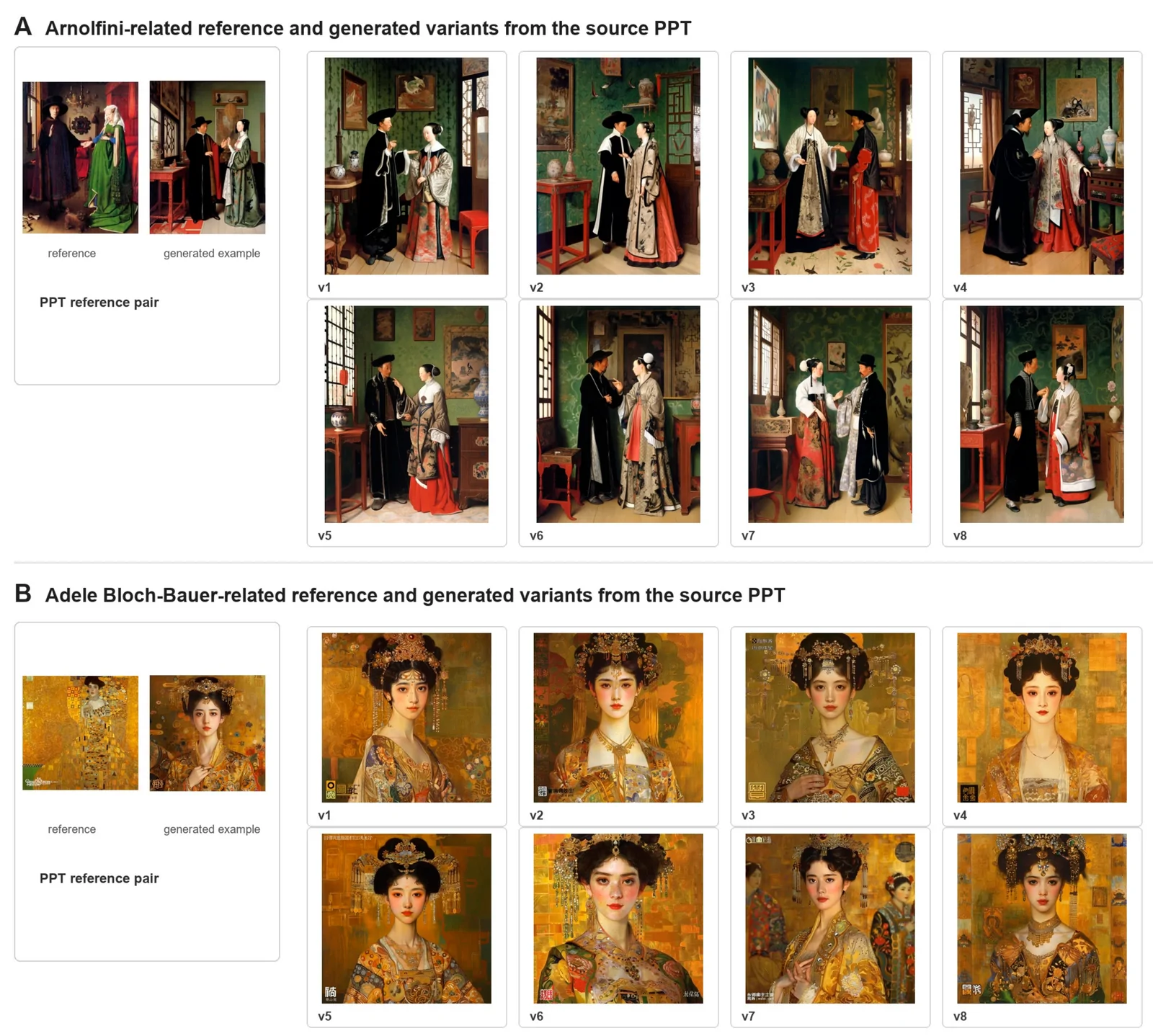
Representative stimulus materials extracted from the supplied presentation file. Panel (**A**) shows an Arnolfini-related reference pair and eight generated variants; Panel (**B**) shows an Adele Bloch-Bauer-related reference pair and eight generated variants. The analysed questionnaire contained five eight-item artwork blocks. The available archive preserves the visual variants used for questionnaire preparation, while incomplete generation metadata and the absence of embedded final images in the SPSS exports are treated as study limitations.

The questionnaire contained five source-painting blocks, each represented by eight AI-generated visual variants: Portrait of Giovanni Arnolfini, Portrait of Adele Bloch-Bauer, The Comtesse d’Haussonville, Charles I Hunting, and The Milkmaid. Thus, participants rated 40 variants within the questionnaire condition to which they were exposed. The available presentation archive preserves the 40 visual variants used to prepare the questionnaire materials, and the available records indicate that the same 40 visual images were used across the three questionnaire versions while the accompanying descriptions varied by cue condition. The SPSS exports preserve the condition-specific wording in variable labels but do not embed the final image files; therefore, the archive supports but cannot by itself independently verify every final image–description page as displayed online. Figure 1 presents the available Arnolfini-related and Adele Bloch-Bauer-related image-series materials extracted from the supplied presentation file; the remaining source-painting blocks are documented in the study inventory and Supplementary Materials rather than reproduced in the main text. The original generation prompts, prompt iterations, Midjourney version information, generation dates, and post-processing history were not fully preserved and are therefore reported as reproducibility limitations rather than reconstructed retrospectively.

The condition manipulation was implemented through Chinese-language descriptions attached to the visual variants. Variable labels in the SPSS exports preserve the wording for each artwork block and condition. The descriptions within a condition expressed a consistent value emphasis while being adapted to the depicted source painting. Table 1 reports translated representative wording from the Arnolfini block. The translation is for reporting purposes; the Chinese wording in the questionnaire export is the auditable stimulus text. Because the cue dimensions necessarily differed in semantic content, the artistic condition was more directly tied to visible formal properties, whereas the cultural and social conditions more often invoked symbolic, historical, or social information. This difference is central to interpretation and is addressed in Discussion as a possible source of the artistic-value advantage.

#### Three-Rater Manipulation Check

To address the manipulation-validation concern raised in prior editorial review, scores from three raters were obtained for a post hoc validation set of 30 image–description pairs covering all five artwork blocks and all three value-cue conditions. The subset was selected to include materials from each source-painting block and each cue condition rather than through a documented random-sampling procedure. It contained 10 artistic, 12 cultural, and 8 social pairs because the available validation file was coverage-oriented rather than proportionally balanced. For each pair, each rater used three 1–7 scales to judge cultural-value alignment, artistic-value alignment, and social-value alignment. These ratings concern cue alignment rather than participants’ aesthetic responses. The scoring file did not identify rater credentials, recruitment procedure, blinding status, or independence. Accordingly, the manipulation check is reported as post hoc representative content-validation evidence, not as exhaustive validation of all image–description pairings or as proof of expert or independent rater status.

The post hoc validation criterion required the mean rater score on an item’s intended value dimension to be at least 5.50 out of 7 and at least 1.00 point higher than its highest mean score on a non-intended dimension. All 30 validation items met this applied criterion. Inter-rater reliability was estimated using atwo-way random-effects, absolute-agreement intraclass correlation coefficient, appropriate when the raters are treated as a sample from a broader rater population (Koo & Li, 2016). Across the 90 item–dimension judgments (30 pairs × 3 value dimensions), reliability was high at ICC(2,1) = 0.830 and ICC(2,k) = 0.936. The intended dimension was also the unique highest-rated dimension for each of the 90 rater-by-item classifications (Table 2).

### 2.3. Procedure

**Figure 2:**
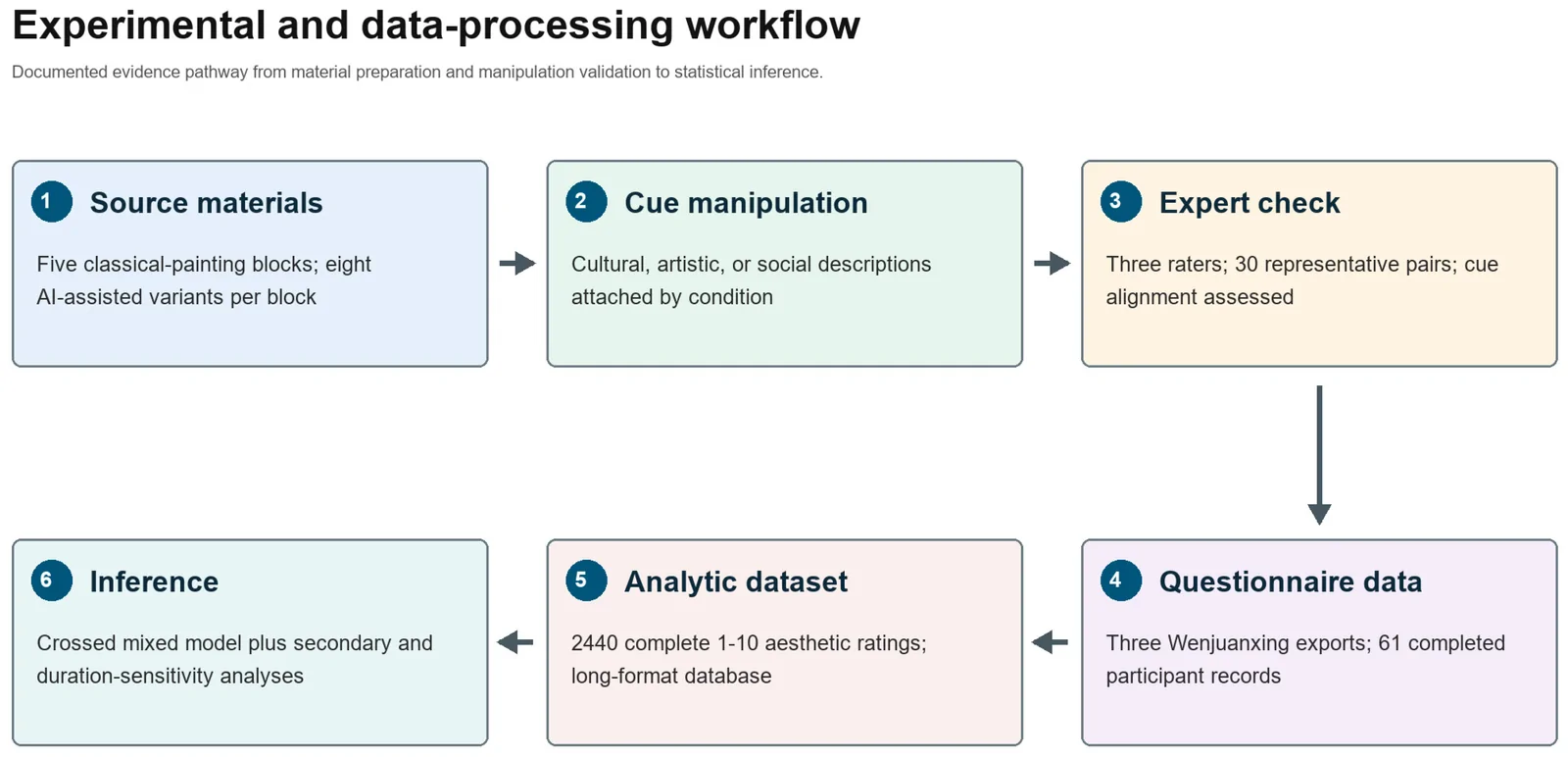
Questionnaireand data-processing workflow. The revised diagram separates material preparation and expert cue-alignment assessment from questionnaire data processing and thecrossed-random-effects inferential analysis, while acknowledging that recruitment and allocation details were not recoverable from the exported files.

According to the questionnaire structure represented in the exports, each participant record comprised one cue-condition version and contained ratings for 40 visual items. Each item combined an image and a value-oriented description before eliciting an aesthetic rating. The exports also contained completion duration and demographic-response fields: gender, age group, ethnicity, and education level. The available files do not document the recruitment invitation, assignment algorithm, IP/device restrictions, or item-randomization settings in Wenjuanxing. Therefore, the revised manuscript no longer treats random assignment or random item order as established facts. Instead, these procedures are identified as undocumented aspects that restrict causal claims and should be prospectively recorded in future replications (Figure 2).

### 2.4. Data Processing and Statistical Analysis

The three SPSS datasets were transformed into a long-format database, with one row per aesthetic rating and identifiers for participant, cue condition, artwork block, and visual variant. Completeness, rating distributions, and completion duration were checked before inferential analysis. Descriptive statistics were calculated for cue conditions and artwork-by-condition cells. No item-level rating was missing. Data processing and statistical analyses were conducted using IBM SPSS Statistics 27.0.

The principal inferential model was a linear mixed-effects model with cue condition as a fixed effect and crossed random intercepts for participant and stimulus identity. This choice recognises that the 40 ratings from each participant are not independent and that visual stimuli are sampled items rather than fixed replications; treating stimuli as random units is important for generalising stimulus-based psychological findings (Barr et al., 2013; Judd et al., 2012). Social-value descriptions were used as the reference condition because the exploratory directional expectations compared cultural and artistic descriptions against social descriptions. In response to review, a fuller model allowing the cultural-versus-social and artistic-versus-social contrasts to vary by stimulus was also estimated. Because this by-stimulus random-slope model converged with a non-positive-definite Hessian, it is reported as a diagnostic model rather than as a stronger basis for expanding the claim.

Secondary analyses were limited to participant-level summaries and sensitivity checks that do not treat the 2440 ratings as independent group observations. These comprised an ANOVA on participant mean ratings, participant-mean pairwise comparisons, descriptive artwork-block summaries, and a post hoc completion-time sensitivity analysis. Rating-level ANOVA, Welch, Kruskal–Wallis, and Tukey tests were removed from the main inferential reporting because their independence assumption is violated by repeated ratings from the same participants and recurrent stimuli. Cronbach’s alpha was calculated for the 40 ratings within each questionnaire version and for each eight-item artwork block. Alpha is reported as response-pattern coherence and not as proof that the stimuli constitute a unidimensional psychological scale, consistent with contemporary cautions about interpreting alpha (Cronbach, 1951; McNeish, 2018). All tests were two-sided.

Completion duration was examined as a data-quality indicator. Nine participants completed the 40-rating questionnaire in less than 60 s. Because the study record did not contain a preregistered duration threshold, no cases were removed from the main analysis. A post hoc sensitivity analysis repeated the participant-level ANOVA and mixed-effects model after omitting these nine records; this analysis is explicitly exploratory and is not used to redefine the primary sample.

The reporting logic distinguishes three evidential levels. First, means, confidence intervals, reliability summaries, and rating-level tests describe the supplied observations and make the dataset auditable. Second, the crossed-random-effects model is the primary inferential analysis because it addresses repeated ratings by participants and recurrence of visual stimuli. Third, the duration exclusion and auxiliary tests are sensitivity or descriptive analyses rather than additional primary hypotheses. This hierarchy prevents a significant result from being selected after inspecting several alternative analyses. It also limits inference to matters supported by the files supplied for revision: the records establish ratings, condition-specific questionnaire exports, and completion times, whereas recruitment, allocation, item-order randomization, unique-participant controls, and expert credentials remain undocumented. A future preregistered replication should specify the primary contrast, stopping rule, exclusion threshold, validation rule, allocation method, and planned model before responses are inspected.

### 2.5. Computational Formalisation of Data and Model Specification

To make the analysis pipeline explicit in formulaic terms, the questionnaire exports can be described as a set of formal relations. Let p index participants, s index visual stimulus items, b index source-artwork blocks, v index generated variants, c index cue condition, and d index the rater-evaluated value dimension. The outcome y_ps_ is the participant’s aesthetic rating of stimulus s on the 1–10 scale. The condition variable c_p_ takes one of three values: social, cultural, or artistic. The following notation states the data structure, post hoc validation criterion, mixed-effects models, and sensitivity set as mathematical expressions.

Formula (1). Long-format rating-data structure.

D = {(p, s, b_s_, v_s_, c_p_, y_ps_): p ∈ P, s ∈ S, c_p_ ∈ C, y_ps_ ∈ {1, …, 10}}, C = {social, cultural, artistic}, |D| = 2440.

The manipulation check can likewise be expressed as a post hoc validation criterion. For each validation item i, the intended value dimension is known from the cue condition. The target score is the mean of the three rater scores on that intended dimension. The non-target score is the larger of the two mean ratings on the remaining dimensions. An evaluated item is classified as meeting the applied criterion when its target mean is at least 5.50 and exceeds the strongest non-target mean by at least one point. This notation describes the validation rule applied to the available ratings and does not imply that the threshold was prospectively documented before the ratings were inspected.

Formula (2). Three-rater cue-alignment validation criterion.

T_i_ = K^−1^ Σ_k_ r_ki_, d(i), K = 3 N_i_ = max d ∈ V, d ≠ d(i) [K^−1^ Σ_k_ r_ki_d] R_i_ = 1{T_i_ ≥ 5.50 ∧ T_i_ − N_i_ ≥ 1.00}.

The principal inferential model can be written as a crossed random-intercept model. Social-value framing is the reference condition, so the cultural and artistic coefficients estimate deviations from social-value framing while participant-specific and stimulus-specific baselines are absorbed as random effects. A fuller diagnostic model additionally allows the cultural-versus-social and artistic-versus-social contrasts to vary by stimulus.

Formula (3). Principal crossed mixed-effects model and diagnostic by-stimulus random-slope extension.

Principal model: y_ps_ = β_0_ + β_1_I(c_p_ = cultural) + β_2_I(c_p_ = artistic) + u_p_ + v_s_ + ε_ps_ u_p_ ∼ N(0, σ^2^_p_), v_s_ ∼ N(0, σ^2^_s_), ε_ps_ ∼ N(0, σ^2^).

Diagnostic by-stimulus random-slope extension: y_ps_ = β_0_ + β_1_I(c_p_ = cultural) + β_2_I(c_p_ = artistic) + u_p_ + v_0s_ + v_1s_I(c_p_ = cultural) + v_2s_I(c_p_ = artistic) + ε_ps_.

The completion-time sensitivity analysis is not a replacement for the primary model. It is a post hoc sensitivity check that removes records below the 60 s threshold and then re-estimates the participant-level and crossed mixed-effects analyses on the reduced dataset.

Formula (4). Completion-time sensitivity sample rule.

P* = {p ∈ P: complete_p_ = 1} P_60_ = {p ∈ P*: t_p_ ≥ 60 s} D_60_ = {(p, s, b_s_, v_s_, c_p_, y_ps_) ∈ D: p ∈ P_60_}.

### 2.6. AI-Assisted Stimulus Production and Reporting Transparency

The source materials identify Midjourney as the tool used to produce AI-assisted stimulus variants from author-designed prompts. AI was used to prepare visual experimental material, not to generate participant responses. Participants evaluated static images and accompanying textual descriptions; their ratings were recorded through the questionnaire platform. The available archive includes representative image-series material and questionnaire-derived variable labels, but it does not contain a complete prompt history, platform-version record, generation log, or post-processing log. These omissions prevent exact reconstruction of the image-generation process and are therefore reported as limits on reproducibility.

The submitted manuscript therefore distinguishes AI as a stimulus-production technology from human participant evaluation and statistical inference. This distinction is important because any cue-condition difference must be interpreted as a response to the visual–textual materials that were actually presented, while acknowledging that undocumented image-selection decisions may have influenced the observed ratings.

## 3. Results

### 3.1. Sample, Descriptive Statistics, and Response Coherence

All 61 completed records contained 40 valid aesthetic ratings. Participant-mean ratings were highest in the artistic-value condition (M = 7.64, SD = 1.78, n = 18), followed by the cultural-value condition (M = 6.83, SD = 1.88, n = 22) and the social-value condition (M = 5.90, SD = 1.83, n = 21). The corresponding rating-level means were identical because each participant contributed 40 ratings, but the confidence intervals in Table 3 are now based on participant means rather than individual ratings. These unadjusted descriptive values establish the direction of differences but do not by themselves address allocation uncertainty or within-stimulus dependence. Demographic fields indicated uneven descriptive composition across questionnaire versions. In the artistic-value version, participants were 11 men and 7 women; age groups were 31–40 years (n = 11) and 26–30 years (n = 7); all identified as Han Chinese; education was undergraduate (n = 8), junior college (n = 5), or postgraduate and above (n = 5). In the cultural-value version, participants were 12 men and 10 women; age groups were 26–30 years (n = 9), 31–40 years (n = 9), 18–25 years (n = 3), and under 18 years (n = 1); ethnicity was Han Chinese (n = 21) or Mongolian (n = 1); education was postgraduate and above (n = 17), undergraduate (n = 2), junior college (n = 1), high school/technical secondary school (n = 1), or junior high school and below (n = 1). In the social-value version, participants were 7 men and 14 women; age groups were 26–30 years (n = 8), 18–25 years (n = 7), 31–40 years (n = 3), over 60 years (n = 2), and 41–50 years (n = 1); ethnicity was Han Chinese (n = 19), Nu (n = 1), or Mongolian (n = 1); education was undergraduate (n = 14), postgraduate and above (n = 3), junior high school and below (n = 2), junior college (n = 1), or high school/technical secondary school (n = 1). These fields are useful for transparency but cannot establish baseline equivalence because recruitment and allocation were not documented. Table 3 summarises the condition-level observations.

Response-pattern coherence was high. Cronbach’s alpha across all 40 ratings was 0.978 in the social-value condition, 0.986 in the cultural-value condition, and 0.984 in the artistic-value condition. Eight-item artwork-block alpha values ranged from 0.931 to 0.989. These values indicate that respondents within each questionnaire version tended to rate the visual items consistently; they should not be interpreted as a validation of a single latent aesthetic-value construct or as a substitute for the expert manipulation check (Table 4).

### 3.2. Three-Rater Manipulation Check

The target-dimension mean across the 30 rater-evaluated pairs was 6.04 out of 7 (SD = 0.24), whereas the highest non-target mean was 2.66 (SD = 0.24), giving a mean separation of 3.39 points (SD = 0.34). Separation was evident for artistic, cultural, and social cue items, and all 30 pairs passed the applied post hoc validation criterion (Table 2). The reliability estimates, ICC(2,1) = 0.830 and ICC(2,k) = 0.936, indicate strong agreement for the representative validation material. This result supports discriminability of the tested cue wording while remaining limited to the non-random, post hoc validation subset.

### 3.3. Primary Mixed-Effects Analysis

The random-intercept mixed-effects model estimated the social-value condition intercept at 5.90 (SE = 0.39). Artistic-value descriptions were associated with ratings 1.74 points higher than social-value descriptions, with b = 1.74, SE = 0.57, z = 3.03, *p* = 0.002, 95% CI [0.62, 2.87]. Cultural-value descriptions were estimated 0.93 points higher than social-value descriptions, but this contrast did not meet the conventional two-sided 0.05 threshold after modelling participant and stimulus variation, with b = 0.93, SE = 0.55, z = 1.70, *p* = 0.088, 95% CI [−0.14, 2.00]. The fuller by-stimulus random-slope model also estimated positive artistic-versus-social and cultural-versus-social contrasts, but it converged with a non-positive-definite Hessian. This diagnostic model therefore does not justify stronger conclusions; it shows only that allowing stimulus-level variation in the condition contrasts did not reverse the narrower artistic-versus-social association (Table 5).

**Figure 3:**
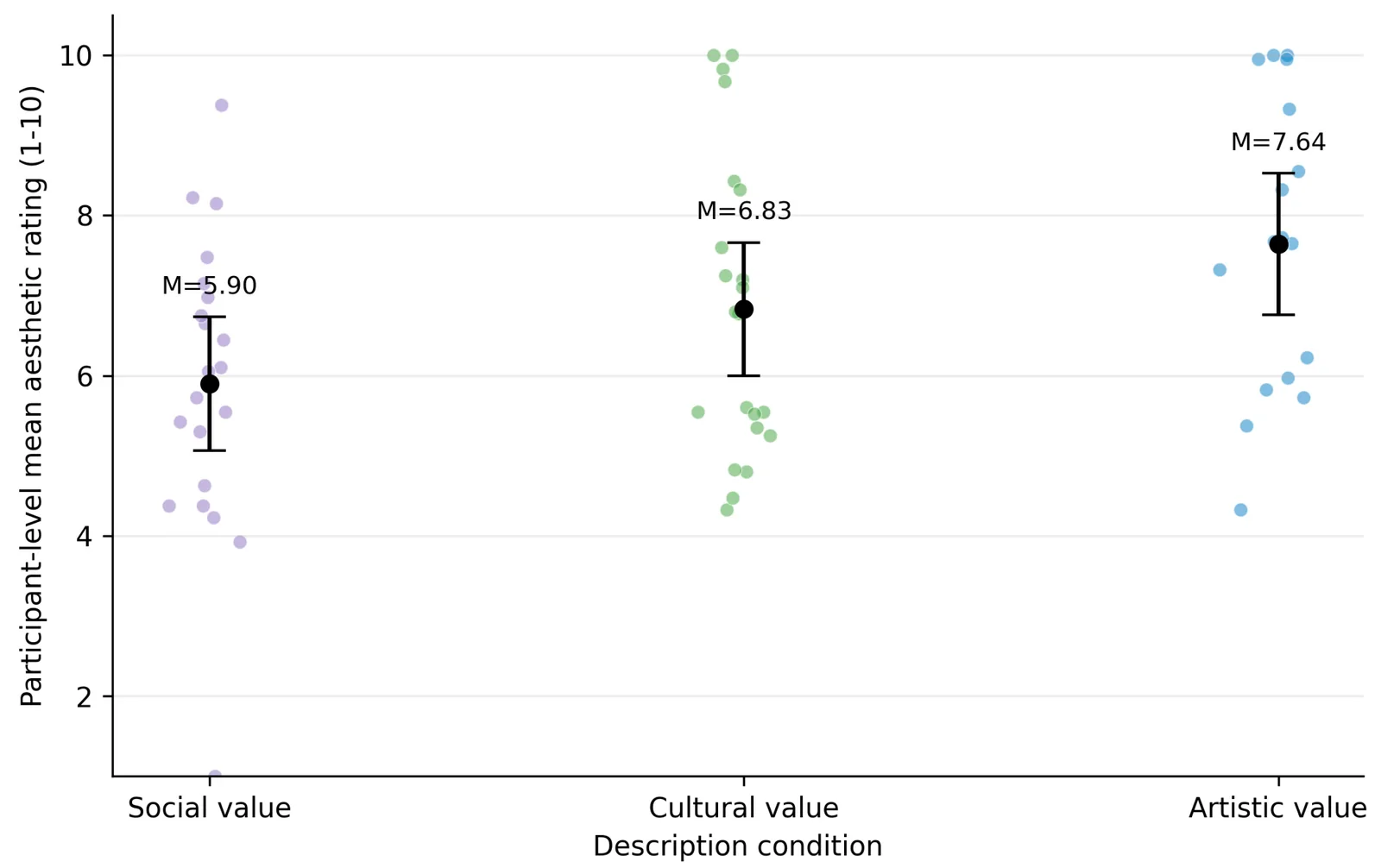
Participant-level mean aesthetic ratings by description cue condition. Points represent participant means; black markers show condition means with 95% confidence intervals.

At the participant-mean level, condition also had an omnibus effect, where F(2, 58) = 4.41, *p* = 0.017, and partial eta squared = 0.132. This analysis treats each participant as one observational unit and therefore provides a complementary check of the principal model, although it does not model stimulus dependence. Participant–mean pairwise comparisons are reported only as exploratory summaries (Table 6). The visual distribution of participant means is shown in Figure 3.

### 3.4. Descriptive Artwork-Block Summaries

The previous rating-level ANOVA, Welch, Kruskal–Wallis, and Tukey tests have been removed from the main inferential reporting because they treated 2440 repeated ratings as independent group observations. The source-artwork block means are retained only as descriptive summaries of the observed rating distribution (Table 4). They show that the artistic-value condition had the highest mean in each of the five artwork blocks, but no rating-level artwork-block moderation test is used as inferential evidence.

Exploratory participant–mean pairwise comparisons were consistent with the central mixed-model pattern: artistic-value participant means were higher than social-value participant means, whereas the cultural-versus-social and artistic-versus-cultural participant-mean comparisons were less certain (Table 6). These comparisons are not a substitute for the mixed-effects models, but they avoid the specific error of treating every rating as an independent participant.

Because artwork block was crossed with participant and stimulus identity, the current revision does not retain the previous rating-level artwork-block ANOVA as an inferential moderation analysis. Descriptively, the ordering of condition means was similar across the five artwork blocks (Table 4), but a confirmatory test of artwork-by-cue moderation would require a prospectively specified mixed-effects model and a larger sample.

### 3.5. Exploratory Completion-Time Sensitivity Analysis

Nine of 61 participants completed all 40 ratings in less than 60 s: two in the artistic-value condition, five in the cultural-value condition, and two in the social-value condition. Their exclusion was not preregistered and therefore was not applied to the main analysis. In an exploratory sensitivity dataset of 52 participants and 2080 ratings, the artistic-value mean remained higher than the social-value mean (Table 7). The participant-level omnibus test was attenuated and no longer conventionally significant, where F(2, 49) = 2.61, *p* = 0.083, and partial eta squared = 0.096. In the random-intercept mixed-effects sensitivity model, the artistic-versus-social contrast persisted, with b = 1.32, SE = 0.59, z = 2.25, *p* = 0.025, 95% CI [0.17, 2.46], while the cultural-versus-social contrast remained uncertain, where b = 0.82, SE = 0.58, and *p* = 0.154. Because the threshold was not prespecified and no attention check was available, this result should be described only as persistence under one post hoc exclusion rule.

Taken together, the analyses answer the exploratory directional expectations unevenly. The clearest evidence concerns E3: artistic-value descriptions received higher aesthetic ratings than social-value descriptions in the principal random-intercept mixed-effects model and persisted under one post hoc completion-time sensitivity analysis. E1 receives descriptive support in the complete sample, but omnibus evidence is less secure after the unplanned duration exclusion and should not be treated as a stable general condition effect. E2 is supported by raw means but does not receive conclusive support in models that accommodate clustering.

## 4. Discussion

### 4.1. Principal Findings

This study investigated whether descriptions emphasising cultural, artistic, or social value were associated with aesthetic ratings of AI-generated variants derived from classical paintings. The clearest finding was that artistic-value descriptions received higher ratings than social-value descriptions. The estimated difference was 1.74 points on a 10-point rating scale in the random-intercept mixed-effects model and persisted at 1.32 points in the exploratory analysis excluding very rapidly completed responses. Cultural-value ratings occupied an intermediate position: they were higher than social-value ratings in raw means, but their mixed-model contrast was uncertain. The evidence therefore supports a differentiated and cautious account of semantic framing, centred primarily on the artistic-versus-social association.

This inference is supported by the manipulation check while remaining proportionate to it. Rater scores clearly separated intended from non-intended value dimensions for the 30 post hoc validation pairs, showing that the selected cue categories were distinguishable as formulated in that subset. The manipulation check does not show that one dimension is inherently more valuable, does not validate every image–description pair used in the questionnaire, and cannot explain why participants produced higher scores. It provides content-validation evidence for the evaluated sample of pairs and supports, but does not exhaustively establish, the manipulation.

### 4.2. Contextual Framing and Aesthetic Cognition

The artistic-value advantage is compatible with models in which a cue facilitates perceptual organisation, attention to relevant features, and interpretation (Chatterjee & Vartanian, 2014; Forster et al., 2013; Leder et al., 2004; Leder & Nadal, 2014; Pelowski et al., 2017; Reber et al., 2004; Skov, 2019). In this experiment, artistic wording referred to visible properties such as light, colour, detail, and composition. Such wording may have supplied an immediately testable interpretive route: participants could compare the described formal quality with the image currently on screen. This may increase processing fluency, strengthen top-down expectations about what should be noticed, or support meaning construction by linking verbal description to observable features. These mechanisms are plausible interpretations rather than directly demonstrated pathways, because the experiment did not separately measure visual attention, perceived fluency, comprehension, individual expertise, or eye movements.

The less stable cultural-value effect also fits prior context research without requiring an unsupported null explanation. Cultural or historical descriptions may invite meaning-making that depends on prior knowledge, interest, or recognition of the referenced tradition (Brieber et al., 2014, 2015; Bullot & Reber, 2013; Darda & Chatterjee, 2023; Kirk et al., 2009; Millis, 2001; Swami, 2013). Social-value descriptions may likewise require viewers to connect the image with broader social relations or public meaning that is not always visible in the stimulus itself. Participants’ art-history familiarity, cultural familiarity, interpretation accuracy, and attitudes toward AI were not available in the supplied analytic records, so the present dataset cannot test whether these variables moderate cultural or social framing.

A further interpretive issue is that the manipulation was not fully isolated from concreteness and perceptual verifiability. Artistic descriptions highlighted formal features that could be checked directly during viewing, whereas cultural and social descriptions invoked background knowledge, rituals, historical position, or social relevance. The observed artistic-value advantage may therefore reflect artistic value, perceptual concreteness, processing fluency, evaluative positivity, ease of verification, or a combination of these factors. The present study cannot adjudicate among these alternatives, and the sections on Discussion and Conclusion have been revised to avoid claiming a demonstrated mechanism.

### 4.3. Contribution to AI-Assisted Art Research

Much recent AI-art research has examined whether an image’s purported human or artificial origin is associated with its evaluation (Bellaiche et al., 2023; Demmer et al., 2023; Grassini & Koivisto, 2024; Horton et al., 2023; Lin et al., 2026; Vessel et al., 2023; Wang et al., 2026; Zhou & Kawabata, 2023). The current study addresses another layer of mediation: even within an AI-assisted image setting, the meaning supplied alongside an image was associated with evaluation. This distinction matters because AI imagery will commonly be encountered within exhibition descriptions, educational explanations, online captions, and curatorial narratives. The present result does not establish guidelines for maximising appreciation, but it indicates that evaluation of AI-assisted images cannot be interpreted independently of their semantic presentation.

The result also suggests a useful methodological role for AI-assisted image families. Multiple variants associated with the same source-artwork blocks permit semantic framing to be examined across recurrent visual identities rather than in a single-image comparison. That advantage depends on transparent generation records. The present archive is incomplete with respect to prompt history, exact model version, generation iterations, selection criteria, and post-processing logs. Consequently, the current study should be read as an analysis of the supplied visual–textual materials rather than as a fully reproducible image-generation protocol.

### 4.4. Methodological Strengths and Limitations

Three features improve the interpretability of the revised analysis. First, the three-rater check directly addresses whether representative cue materials express the intended value categories. Second, the primary model acknowledges dependence among repeated participant ratings and recurrent stimulus identities rather than relying on item-level ANOVA. Third, the exploratory duration sensitivity analysis exposes how a plausible data-quality concern affects the estimated pattern: it reduces omnibus certainty while leaving the principal artistic-social contrast present under one post hoc exclusion rule.

The limitations are nevertheless consequential. The total sample was modest and group sizes were unequal. The available exports do not document recruitment, condition allocation, unique-participant safeguards, or presentation-order randomization, although an earlier draft described these procedures. The study therefore does not establish random assignment, comparable baseline groups, or protection against self-selection into questionnaire versions. It also contains no verified measure of participants’ expertise, familiarity with the paintings, attitudes toward AI, or awareness of how the images were made. Any of these variables might affect appraisal or interaction with the contextual text.

Stimulus validation is also incomplete in scope. Thirty image–description pairs were differentiated by the three raters, but the validation file does not identify rater expertise or blinding, and it does not establish validation of every rated image–description pairing. The validation subset was coverage-oriented rather than randomly sampled or proportionally balanced across cue conditions. The description conditions further contain differences in semantic content that are intrinsic to the intended manipulation; a future study should additionally match length, valence, complexity, factual specificity, and perceptual verifiability, and should include manipulation-check questions administered independently of the aesthetic outcome.

Finally, the completion-time issue is substantial. Nine of 61 participants completed all 40 ratings in less than 60 s, which is much shorter than the 10–15 min duration described in the consent materials. Because the threshold was not preregistered, excluding these cases cannot be treated as a definitive correction, but retaining them also leaves a meaningful data-quality concern. The exploratory exclusion removes participant-level omnibus significance while preserving only the artistic-versus-social mixed-model contrast. Accordingly, the revised Abstract, Results, Discussion, and Conclusion emphasise the artistic-value contrast rather than a broad and stable effect of all cue conditions.

Rating-level inferential analyses were removed from the main text because repeated observations violate the independence assumption of those tests. The participant-level and mixed-effects findings provide a more defensible foundation for inference, but larger preregistered studies are needed to estimate cultural–social effects precisely, test variation by artwork and expertise, and compare AI-assisted with original or human-edited stimuli. As the questionnaire is worded in Chinese while the image sources are canonical Western paintings, replication across languages, cultural settings, and art traditions is also important.

### 4.5. Reproducibility, Documentation, and Ethical Accountability

The present findings also illustrate why semantic-framing studies involving AI-assisted art require a reporting standard beyond conventional stimulus naming. A cue association may arise from the interpretive information accompanying an image, from visual properties of the selected variant, from participants’ assumptions about generative AI, or from interactions among these factors. Reproducible interpretation therefore depends on retaining the exact questionnaire wording in its original language, translated reporting text, a stable identifier for each presented image, the generation and selection record for the AI-assisted variants, and the mapping from each image to its cue condition. The current archive preserves the visual variants used for questionnaire preparation and the condition-specific wording in SPSS labels, but the absence of embedded final questionnaire images, complete prompt histories, Midjourney version information, and generation logs remains a reproducibility limitation. The post hoc three-rater check improves content validity for the available validation set, but a confirmatory replication should validate the full stimulus set using raters whose qualifications, independence, instructions, and blinding status are disclosed.

The same principle applies to analysis decisions. The artistic-versus-social contrast persisted within the supplied sample in the random-intercept mixed-effects model and after one exploratory duration screen. It should nevertheless be tested prospectively with a sample-size justification, randomised or otherwise documented condition allocation, prespecified attention and duration rules, and a primary model defined before data inspection. A replication should also measure art expertise, prior familiarity with each source painting, attitudes toward AI-generated imagery, and comprehension of the descriptive cue. These variables would make it possible to test whether artistic descriptions operate through accessible visual features, whether cultural cues depend on background knowledge, or whether AI-related expectations moderate either pathway. Without these measures, the present psychological account remains a theoretically informed interpretation of a narrow condition association rather than a demonstrated mechanism.

Ethical documentation is part of this evidentiary standard rather than a separate administrative afterthought. The study used a participant-facing informed-consent form that described the academic purpose of the research, voluntary participation and withdrawal, low-risk visual-rating procedures, data privacy, aggregate reporting, and the use of AI-assisted image materials. The analytic records are anonymous and contain no directly identifying information. Preserving the consent record, de-identified dataset, analysis syntax, and stimulus archive strengthens accountability and makes the study more reusable for cumulative research on aesthetic judgement in AI-mediated settings.

## 5. Conclusions

Across 61 completed participant records and 2440 aesthetic ratings, the defensible central finding is that artistic-value descriptions were associated with higher ratings than social-value descriptions after modelling participant and stimulus variation, and that this contrast persisted under one exploratory completion-time sensitivity analysis. Cultural-value descriptions were intermediate, but their advantage over social-value descriptions was not conclusive in the primary model. Rater scores supported the discriminability of the post hoc validation subset. The study therefore contributes qualified, exploratory evidence that semantic framing oriented toward visible artistic execution is associated with aesthetic appraisal of AI-assisted imagery. Because recruitment, assignment, complete stimulus-generation records, and the full validation history were not recoverable, broader causal claims require complete procedural documentation and larger preregistered replication.

## Figures and Tables

**Table 1:** Value-description manipulation and representative translated wording from the Arnolfini block.

Cue Type	Operational Emphasis	Representative Questionnaire Wording Translated from Chinese
Cultural value	Tradition and symbolic meaning	The image depicts a couple’s life setting, reflecting marriage rituals and family ideas within a cultural tradition and symbolising family harmony and fidelity.
Artistic value	Technique and visual execution	The image employs delicate technique and finely rendered details, displaying skillful control of light and shadow; its colour coordination and composition possess artistic appeal.
Social value	Historical social significance	In the social context of its time, the image was significant, reflecting the importance attached to marriage and family in a particular historical period and exerting influence in the art world.

**Table 2:** Three-rater manipulation-check summary for the post hoc validation set.

Cue Condition	n Pairs	Target M (SD)	Highest Non-Target M (SD)	Difference M (SD)	Met Applied Post Hoc Criterion
Artistic value	10	6.07 (0.26)	2.63 (0.25)	3.43 (0.39)	10 (100.0%)
Cultural value	12	6.14 (0.22)	2.70 (0.22)	3.44 (0.30)	12 (100.0%)
Social value	8	5.88 (0.17)	2.62 (0.28)	3.25 (0.35)	8 (100.0%)
Overall	30	6.04 (0.24)	2.66 (0.24)	3.39 (0.34)	30 (100.0%)

**Table 3:** Descriptive statistics for aesthetic ratings by cue condition.

Cue Condition	Participants	Ratings	Participant Mean	Participant-Level SD	95% CI of Participant Mean
Social value	21	840	5.90	1.83	[5.06, 6.73]
Cultural value	22	880	6.83	1.88	[6.00, 7.66]
Artistic value	18	720	7.64	1.78	[6.76, 8.53]

**Table 4:** Descriptive statistics by source-artwork block and cue condition.

Artwork Block	Cue Condition	Mean	SD	n Ratings
Portrait of Giovanni Arnolfini	social value	5.40	2.27	168
Portrait of Giovanni Arnolfini	cultural value	6.47	2.52	176
Portrait of Giovanni Arnolfini	artistic value	7.03	2.48	144
Portrait of Adele Bloch-Bauer	social value	6.15	2.69	168
Portrait of Adele Bloch-Bauer	cultural value	7.12	2.02	176
Portrait of Adele Bloch-Bauer	artistic value	7.65	2.31	144
The Comtesse d’Haussonville	social value	6.12	2.38	168
The Comtesse d’Haussonville	cultural value	6.78	2.26	176
The Comtesse d’Haussonville	artistic value	7.78	2.06	144
Charles I Hunting	social value	5.41	2.36	168
Charles I Hunting	cultural value	6.80	2.27	176
Charles I Hunting	artistic value	7.55	2.49	144
The Milkmaid	social value	6.41	2.63	168
The Milkmaid	cultural value	6.97	2.39	176
The Milkmaid	artistic value	8.20	1.84	144

**Table 5:** Mixed-effects and participant-level analyses for the complete sample.

Analysis	Statistic	*p*-Value	Effect Size or Estimate
Random-intercept mixed model: artistic vs. social	b = 1.74, SE = 0.57, z = 3.03	0.002	95% CI [0.62, 2.87]
Random-intercept mixed model: cultural vs. social	b = 0.93, SE = 0.55, z = 1.70	0.088	95% CI [−0.14, 2.00]
By-stimulus random-slope model: artistic vs. social	b = 1.74, SE = 0.14, z = 12.46	Diagnostic only	95% CI [1.47, 2.02]; Hessian non-positive definite
By-stimulus random-slope model: cultural vs. social	b = 0.93, SE = 0.15, z = 6.24	Diagnostic only	95% CI [0.64, 1.22]; Hessian non-positive definite
Participant-level ANOVA	F(2, 58) = 4.41	0.017	partial eta squared = 0.132

**Table 6:** Exploratory participant–mean pairwise comparisons.

Comparison	Mean Difference	95% CI	*p*-Value
Artistic value–social value	1.74	[0.55, 2.93]	0.0048
Cultural value–social value	0.93	[−0.21, 2.07]	0.108
Artistic value–cultural value	0.81	[−0.37, 2.00]	0.169

**Table 7:** Exploratory sensitivity analysis excluding participants with completion duration below 60 s.

Cue Condition	Participants Retained	Ratings	Participant Mean (SD)	Mixed-Model Contrast vs. Social
Social value	19	760	6.03 (1.44)	Reference
Cultural value	17	680	6.86 (2.05)	b = 0.82, *p* = 0.154
Artistic value	16	640	7.35 (1.67)	b = 1.32, *p* = 0.025

## Data Availability

The de-identified questionnaire dataset, expert-rating data, and analysis materials are available from the corresponding author upon reasonable request, subject to participant-consent and institutional requirements.

## Supplementary Materials

The following supporting information can be downloaded at https://www.mdpi.com/article/10.3390/bs16081437/s1, File S1: Stimulus, Procedure, and Transparency Documentation.
