# Supplementary material for: Artistic-Value Descriptions Are Associated with Higher Aesthetic Ratings than Social-Value Descriptions for AI-Generated Variants of Classical Paintings
Source: Behav Sci (Basel). 2026 Aug 20;16(8):1437. doi: 10.3390/bs16081437 (PMC13509515; doi:10.3390/bs16081437)
Supplement: Supplementary file 1 [file behavsci-16-01437-s001.zip › behavsci-4376768-supplementary.pdf]

Supplementary File S1

Stimulus, Procedure, and Transparency Documentation

**Manuscript ID**	behavsci-4376768
**Journal**	Behavioral Sciences
**Manuscript title**	Cultural, Artistic, and Social Value Cues Shape Aesthetic Evaluations of AI-Generated Variants of Classical Paintings

Purpose

This supplementary documentation summarizes the materials available for editorial review and clarifies the limits of the archived stimulus-generation record.

Available Study Materials

Material	Available documentation	Reproducibility note
Questionnaire data	Three Wenjuanxing/SPSS export files corresponding to artistic-, cultural-, and social-value questionnaire versions.	Documents completed records, item ratings, and completion time.
Visual stimuli	A source presentation file containing representative image-series materials, including Arnolfini-related and Adele Bloch-Bauer-related blocks.	The full generation history was not preserved in the exported analytic files.
Cue wording	Chinese questionnaire variable labels and translated representative wording in the manuscript.	Original Chinese wording is the auditable stimulus text.
Expert validation	Expert-rating scores for 30 representative image-description pairs and recomputed summary files.	Representative validation only; not exhaustive full-set validation.
Ethics/consent	Participant informed-consent documentation and institutional ethics exemption statement.	Supports ethical documentation and informed consent.

Stimulus Blocks

The questionnaire contained five source-painting blocks, each represented by eight AI-generated variants: Portrait of Giovanni Arnolfini, Portrait of Adele Bloch-Bauer, The Comtesse d'Haussonville,

Charles I Hunting, and The Milkmaid. Participants rated 40 image-description items within the questionnaire version to which they were exposed.

AI-Assisted Generation Record

The available source materials identify Midjourney as the image-generation platform. The exact Midjourney model/version, full prompt history, prompt iterations, generation dates, output selection criteria, and post-processing history were not fully preserved in the archived analytic exports. These items are therefore not reconstructed retrospectively and are reported in the manuscript as reproducibility limitations.

Recommended Use

This file should be read together with the revised manuscript, the available stimulus source presentation, expert-validation files, and questionnaire exports. The authors do not claim that this documentation enables exact regeneration of the images; rather, it documents the materials available for editorial assessment and future methodological transparency.

Block-Level Stimulus Inventory

Source-painting block	Presented variants	Main-text visual documentation
Portrait of Giovanni Arnolfini	Eight AI-generated variants	Representative materials shown in Figure 1
Portrait of Adele Bloch-Bauer	Eight AI-generated variants	Representative materials shown in Figure 1
The Comtesse d'Haussonville	Eight AI-generated variants	Documented in study inventory/source materials; not reproduced in main text
Charles I Hunting	Eight AI-generated variants	Documented in study inventory/source materials; not reproduced in main text
The Milkmaid	Eight AI-generated variants	Documented in study inventory/source materials; not reproduced in main text

This inventory is intended to clarify the block structure of the questionnaire. It does not claim that the full AI-generation process can be exactly regenerated, because the complete prompt and model-version history was not preserved.
